# Structure and transport mechanism of the human calcium pump SPCA1

**DOI:** 10.1038/s41422-023-00827-x

**Published:** 2023-05-31

**Authors:** Mengqi Wu, Cang Wu, Tiefeng Song, Kewu Pan, Yong Wang, Zhongmin Liu

**Affiliations:** 1 Department of Immunology and Microbiology, School of Life Sciences, Southern University of Science and Technology, Shenzhen, Guangdong China; 2 College of Life Sciences, Zhejiang University, Hangzhou, Zhejiang China; 3 School of Biomedical Sciences, The Chinese University of Hong Kong, Hong Kong, China; 4 The Provincial International Science and Technology Cooperation Base on Engineering Biology, International Campus of Zhejiang University, Haining, Zhejiang China

**Keywords:** Cryoelectron microscopy, Molecular biology

## Abstract

Secretory-pathway Ca^2+^-ATPases (SPCAs) play critical roles in maintaining Ca^2+^ homeostasis, but the exact mechanism of SPCAs-mediated Ca^2+^ transport remains unclear. Here, we determined six cryo-electron microscopy (cryo-EM) structures of human SPCA1 (hSPCA1) in a series of intermediate states, revealing a near-complete conformational cycle. With the aid of molecular dynamics simulations, these structures offer a clear structural basis for Ca^2+^ entry and release in hSPCA1. We found that hSPCA1 undergoes unique conformational changes during ATP binding and phosphorylation compared to other well-studied P-type II ATPases. In addition, we observed a conformational distortion of the Ca^2+^-binding site induced by the separation of transmembrane helices 4L and 6, unveiling a distinct Ca^2+^ release mechanism. Particularly, we determined a structure of the long-sought CaE2P state of P-type IIA ATPases, providing valuable insights into the Ca^2+^ transport cycle. Together, these findings enhance our understanding of Ca^2+^ transport by hSPCA1 and broaden our knowledge of P-type ATPases.

## Introduction

Calcium ion (Ca^2+^) is a vital signaling molecule in cells that regulates numerous cellular processes, such as proliferation, differentiation, apoptosis, secretion, contraction, and fertilization.^1^ The Golgi apparatus (GA), along with the endoplasmic reticulum (ER) and mitochondria, serve as critical Ca^2+^ storage organelles that regulate Ca^2+^ homeostasis and subsequent Ca^2+^ signaling in cells.^2^ Maintaining an appropriate Ca^2+^ concentration in the GA lumen is critical for normal protein synthesis, processing, and sorting.^3^ Several proteins, including sarcoplasmic/endoplasmic reticulum Ca^2+^-ATPases (SERCAs), inositol-1,4,5-trisphosphate receptors (IP_3_Rs), ryanodine receptors (RYRs), and secretory pathway Ca^2+^-ATPases (SPCAs), play essential roles in balancing the Ca^2+^ homeostasis of GA.^2,4^ While the transport mechanisms of SERCAs,^5–7^ IP_3_Rs,^8,9^ and RYRs^10–13^ have been well structurally characterized using X-ray crystallography and single-particle cryo-electron microscopy (cryo-EM), the transport mechanism of SPCAs remains elusive due to a lack of crucial structural information. Moreover, unlike other Ca^2+^ transporters distributed on multiple organelles,^4^ SPCAs are mainly localized to the GA^3,14^ and may play unique roles in regulating the functions of GA.

The SPCA family in mammals consists of at least two subtypes, SPCA1 and SPCA2, which are encoded by the *ATP2C1* and *ATP2C2* genes, respectively.^3,4^ SPCA1 was identified as a Ca^2+^ pump earlier than SPCA2,^15^ and as a result, its physiological function and properties are better understood. SPCA1 is crucial for maintaining the proper structure and function of the GA.^14,16^ Silencing of SPCA1 induces fragmentation of the GA ribbon and slows down protein transport in the Golgi compartments.^14^ In human epidermal keratinocytes, SPCA1 contributes ~67% of Ca^2+^ uptake into the GA, making keratinocytes highly sensitive to changes in functional SPCA1 level.^3,17^ Haploinsufficiency of SPCA1 leads to the development of Hailey-Hailey disease (HHD), a heritable autosomal dominant skin disease.^18^ Deficiency of SPCA1-mediated Ca^2+^ transport appears to be the causative factor of HHD.^19,20^ Additionally, SPCA1 has been implicated in basal-type breast cancer,^21^ and its upregulation is associated with microcalcifications during breast cancer development,^22,23^ a process dependent on SPCA1’s Ca^2+^-pumping activity.^22^ Therefore, a thorough characterization of SPCA1-mediated Ca^2+^ transport may provide novel insight into understanding diseases associated with SPCA1.

SPCA1 is a member of the P-type IIA ATPases^24^ and transports substrates through a series of intermediate states in a process known as the Post-Albers reaction cycle.^25^ This cycle includes several intermediate states such as CaE1, CaE1-ATP, CaE1P-ADP, CaE1P, CaE2P, E2P, E2~P, E2Pi, E2, and E1 (where P indicates protein phosphorylation and Pi indicates non-covalently bound phosphate).^26–28^ It is worth noting that CaE1P is an ADP-sensitive state in which the phosphorylated Asp is far away from the TGE motif, allowing the binding of ADP to regenerate ATP in a reverse reaction.^29,30^ Due to the loss of ADP binding affinity at the catalytic site, CaE2P is believed to be an ADP-insensitive state with occluded Ca^2+^ at the transport site.^29,31^ E2P is an intermediate state after Ca^2+^ release.^28^ SERCAs, also members of P-type IIA ATPases, have been extensively studied and serve as a model system for understanding the Ca^2+^ transport mechanism of P-type Ca^2+^-ATPases.^32,33^ However, there are some key differences between SERCAs and SPCA1. For example, SERCAs contain two high-affinity Ca^2+^-binding sites, namely site I and site II, and transport two Ca^2+^ per ATP hydrolysis cycle,^5,6^ whereas SPCA1 may transport only one divalent cation per Post-Albers cycle.^34^ Additionally, antagonists that effectively inhibit SERCAs, such as thapsigargin (TG) and cyclopiazonic acid (CPA), show weaker inhibitory effects on SPCA1,^35,36^ suggesting a distinct inhibitory mechanism. Notably, there is still no structure in the CaE2P state available even for the most extensively studied SERCAs, limiting our understanding of the complete Ca^2+^ transport cycle of P-type IIA ATPases. Recently, the structures of hSPCA1 in the CaE1-ATP and E2P states were reported, providing detailed insights into ATP and Ca^2+^ binding as well as some information on the structural basis for Ca^2+^ release.^37^ However, the current structural information is insufficient to fully explain the complete Ca^2+^ transport mechanism of hSPCA1.

In this study, we determined the cryo-EM structures of hSPCA1 in six intermediate states, which allowed us to characterize the Ca^2+^-binding pocket and capture the conformational rearrangements of hSPCA1 throughout a near-complete Post-Albers cycle. Further all-atom molecular dynamics (MD) simulations with explicit solvent enabled us to identify functional states and capture substrate entry and exit pathways. Our results provide unprecedented insight into the Ca^2+^ transport mechanism of hSPCA1, expand our understanding of the working mode of P-type II ATPases, and advance our knowledge of P-type ATPases.

## Results

### Structure determination and overall architecture of hSPCA1

To study the structure of hSPCA1, we isolated and purified the hSPCA1 proteins (see more details in Materials and Methods). Analysis using size-exclusion chromatography (SEC) and sodium dodecyl sulfate-polyacrylamide gel electrophoresis (SDS-PAGE) showed that hSPCA1 was highly pure and eluted as a single and symmetric peak (Supplementary information, Fig. S1a, b). Western blot assay confirmed the identity of the protein (Supplementary information, Fig. S1c), and an ATPase activity assay demonstrated that it has ATP hydrolysis activity (Supplementary information, Fig. S1d). These results indicated that the purified hSPCA1 was qualified for cryo-EM analysis.

**Fig. 1:**
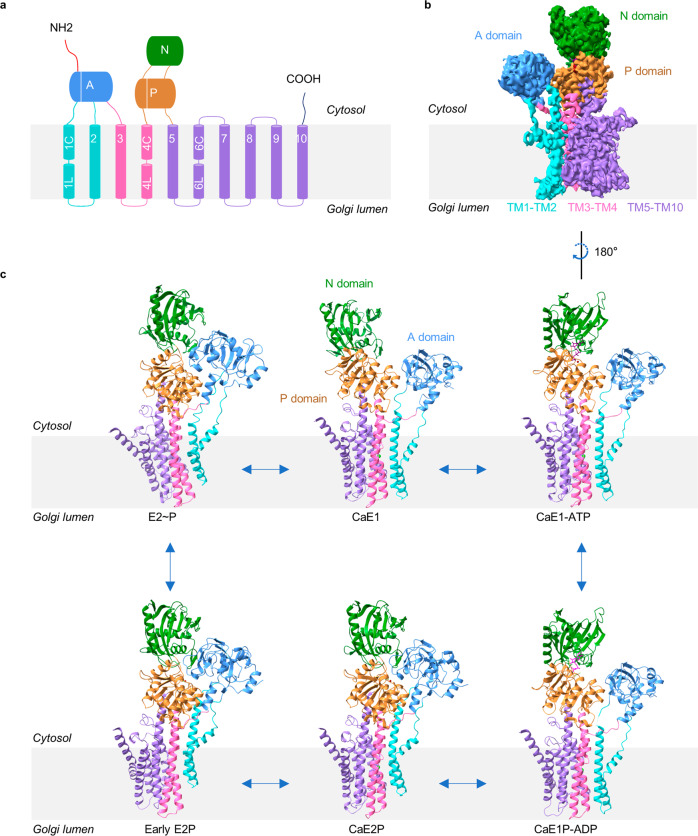
Overall architecture of hSPCA1. **a** Topology diagram of hSPCA1. Conserved cytoplasmic domains and TM helices are schematically illustrated. The domains are colored as follows: A domain, cornflower blue; N domain, forest green; P domain, peru (orange-brown); N-terminal region, red; C-terminal region, deep blue; TM1–TM2, cyan; TM3–TM4, hot pink; TM5–TM10, medium purple. **b** Cryo-EM map (contoured at 5.3 σ) of hSPCA1 in the CaE1-ATP state, shown with the same color scheme as in **a**. **c** Atomic models of the six resolved structures of hSPCA1.

To gain a detailed understanding of the transport cycle of hSPCA1, we incubated the protein with different ATP or phosphate analogs before preparing cryo-EM samples using methods described in previous studies (see more details in Materials and Methods).^6,29,38,39^ We recorded and processed movies of hSPCA1 in different states using cryoSPARC^40^ and Relion^41^ softwares (Supplementary information, Figs. S2–S5), and obtained cryo-EM structures representing six intermediate states (Fig. 1c) at overall resolutions ranging from 3.2 Å to 3.7 Å according to the gold-standard Fourier shell correlation (FSC) 0.143 criterion (Supplementary information, Figs. S2–S5 and Table S1). This allowed us to build accurate atomic models of most regions of hSPCA1 (except the two flexible terminals, Met1 to Glu15, and Gln906 to Val919) and to assign the structures to specific intermediate states in the transport cycle (Fig. 1c). We first focused on the AMP-PCP-bound structure in the CaE1-ATP state to characterize the overall structure of hSPCA1.

**Fig. 2:**
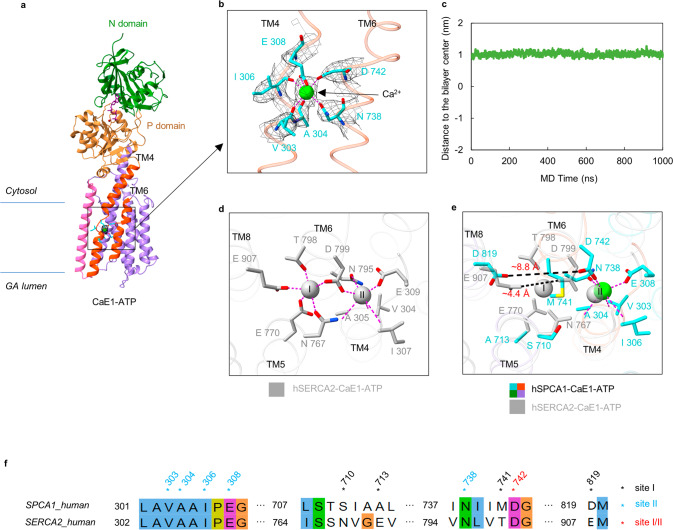
Structural basis for Ca^2+^ binding. **a** Side view of hSPCA1 in the CaE1-ATP state. N and P domains are colored forest green and peru, respectively. A domain and TM1–TM2 are hidden for a clearer view. TM3 is colored hot pink. TM4 and TM6 are colored orange-red. TM5 and TM7–TM10 are medium purple. Ca^2+^ is shown as the lime sphere. The Ca^2+^ density (contoured at 15 σ) is shown as black mesh. **b** Atomic models and cryo-EM densities of Ca^2+^ and Ca^2+^-binding site of hSPCA1 in the CaE1-ATP state. Ca^2+^-binding residues are shown as the cyan sticks and Ca^2+^ is shown as the lime sphere. The densities are shown as black mesh, contoured at 15 σ. The interactions between residues and Ca^2+^ are shown by magenta dashed lines. **c** The stability of the Ca^2+^ substrate in the binding site observed in a 1-µs explicit solvent all-atom MD simulation. It shows the trajectory of the distance between the Ca^2+^ ion and the center of the lipid bilayer. **d** The two Ca^2+^-binding sites of hSERCA2 in the CaE1-ATP state (PDB: 6LLE). Ca^2+^ ions are shown as dark gray spheres. Ca^2+^-binding residues are shown as sticks. The magenta dashed lines represent the metal coordination bonds. **e** Comparison of the Ca^2+^-binding sites between hSPCA1 and hSERCA2 in the CaE1-ATP state. Site I and site II counterpart residues in hSPCA1 are shown as cyan sticks. Dark gray sticks are the Ca^2+^-binding site I residues in hSERCA2. The black dashed lines show the distance between the Ca^2+^-binding oxygen atoms from Asp799 and Glu907 in hSERCA2 and the distance between the oxygen atoms from Asp742 and Asp819 in hSPCA1, respectively. **f** Sequence alignment of hSPCA1 and hSERCA2. Black asterisks indicate residues contributing to Ca^2+^-binding site I. Blue asterisks indicate residues contributing to Ca^2+^-binding site II. The red asterisk indicates the residue contributing to both sites I and II.

**Fig. 3:**
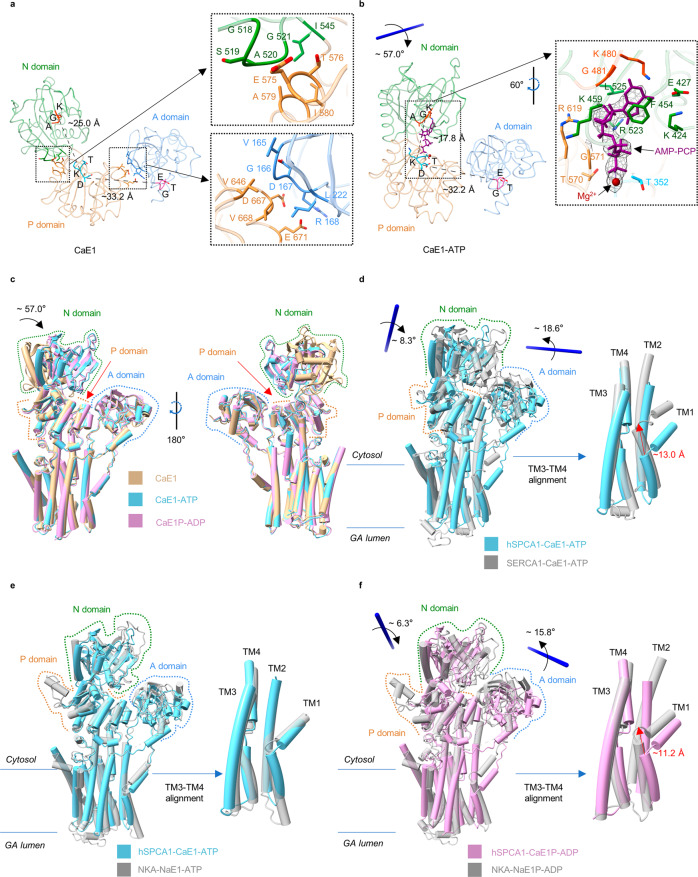
A unique movement of cytosolic domains and TMD during phosphorylation. **a** Cytoplasmic domains of hSPCA1 in the CaE1 state. A, N, and P domains are colored cornflower blue, forest green, and peru, respectively. Conserved ^189^TGE, ^480^KGA, and ^350^DKT motifs are colored hot pink, orange-red, and cyan, respectively. The dashed lines in the left inset show the distances from the Cα of Gly481 to the Cα of Lys351 and from the Cα of Glu191 to the Cα of Asp350. The right upper inset and right lower inset show the interaction surface between domains N and P, and that between domains A and P, respectively. The residues are shown as sticks. **b** Cytoplasmic domains of hSPCA1 in the CaE1-ATP state. AMP-PCP (purple ribbon), Mg^2+^ (dark red sphere), and the corresponding cryo-EM densities (black mesh, contoured at 16.8 σ) are shown. The dashed lines in the left inset show the distances from the Cα of Gly481 to the Cα of Lys351, and from the Cα of Glu191 to the Cα of Asp350. **c** Structural comparison of states CaE1 (tan), CaE1-ATP (light sky blue) and CaE1P-ADP (plum) of hSPCA1 by global alignment. **d** Structural comparison by P domain alignment (left) and TM3–TM4 alignment (right) of hSPCA1 (light sky blue) and SERCA1 (dark gray, PDB: 1T5S) in the CaE1-ATP state. The red arrow (right) shows the distance from the Cα of Pro82 in hSPCA1 to the Cα of Leu60 in SERCA1. **e** Structural comparison by global alignment (left) and TM3–TM4 alignment (right) of hSPCA1 (light sky blue) and NKA (dark gray, PDB: 7E21) in the substrate-bound E1-ATP state. **f** Structural comparison by P domain alignment (left) and TM3–TM4 alignment (right) of hSPCA1 (plum) and NKA (dark gray, PDB: 8D3U) in the substrate-bound E1P-ADP state. The red arrow (right) shows the distance from the Cα of Pro82 in hSPCA1 to the Cα of Gly89 in NKA.

The overall structure of hSPCA1 is typical of a P-type ATPase, with ten transmembrane (TM) helices (TM1–TM10) and three conserved cytosolic domains: the actuator (A), phosphorylation (P), and nucleotide-binding (N) domains (Fig. 1a–c). The A domain is connected to TM1–TM3, while the P domain is linked to TM4 and TM5. The N domain is connected to the P domain through two linkers (Fig. 1a–c). Notably, TM1, TM4, and TM6 are divided into two sub-helices (TM1C and TM1L, TM4C and TM4L, TM6C and TM6L, C indicated the cytosol and L represented the GA lumen) (Fig. 1a; Supplementary information, Fig. S6a). TM2–TM5 have long cytoplasmic extensions (Fig. 1c; Supplementary information, Fig. S6a). Three characteristic cytosolic amino acid motifs in P-type ATPase (^480^KGA in the N domain for ATP binding, ^350^DKT in the P domain for phosphorylation, and ^189^TGE in the A domain for dephosphorylation)^42^ were well solved in the EM structure (Supplementary information, Fig. S6b). An AMP-PCP molecule was observed near the conserved ^480^KGA and ^350^DKT motifs, interacting with an Mg^2+^ ion and several residues, including Thr352, Lys424, Glu427, Phe454, Lys459, Lys480, Gly481, Arg523, Leu525, Thr570, Gly571, and Arg619 (Fig. 3b), similar to the reported CaE1-ATP structure.^37^ Additionally, a well-defined Ca^2+^ density was observed in the TM domain (TMD) (Fig. 2a, b; see the next section for more details). These structural features allowed us to assign the AMP-PCP-bound structure of hSPCA1 to the CaE1-ATP state.

### Structural basis for Ca^2+^ binding

To gain insight into how hSPCA1 recognizes Ca^2+^, we successfully obtained the structures of hSPCA1 bound to Ca^2+^ in three different states: CaE1, CaE1-ATP, and CaE1P-ADP. These structures had resolutions of 3.59 Å, 3.52 Å, and 3.71 Å, respectively (Supplementary information, Figs. S2, S3). We found that the TMDs were nearly identical in all three structures (Fig. 3c). Additionally, we observed an ion-like EM density between TM4 and TM6 (Fig. 2a, b; Supplementary information, Fig. S6d), surrounded by highly conserved residues across the SPCA family (Supplementary information, Fig. S6c). Based on the biochemical assay^43^ and 1-µs explicit solvent all-atom MD simulations of the CaE1-ATP structure (Fig. 2c), we identified this density as a Ca^2+^ ion that is stably bound to the protein.

Structural analysis revealed that the Ca^2+^ coordinates with the side-chain oxygen atoms of several residues, including Glu308, Asn738, and Asp742, and the main-chain carbonyl oxygen atoms of other residues, including Val303, Ala304, and Ile306 (Fig. 2b), consistent with the results reported by Chen et al.^37^ We also found that the Ca^2+^-binding site in hSPCA1 corresponds to site II in hSERCA2 (Fig. 2d, e). However, hSPCA1 does not have a corresponding site I because some related residues (e.g., Asn767 and Glu770 in hSERCA2) are replaced by smaller ones (Ser710 and Ala713 in hSPCA1) and the potential Ca^2+^-binding site is further occupied by a bulky residue Met741 in hSPCA1 (Fig. 2d–f). Asp799 in TM6 and Glu907 in TM8 coordinate with the Ca^2+^ in the site I of hSERCA2 with a distance of ~4.4 Å between the two residues (Fig. 2d, e), but the counterpart distance (Asp742 and Asp819) in hSPCA1 is ~8.8 Å (Fig. 2e), too large to coordinate with a Ca^2+^ ion. This disrupts the coordination geometry of the potential Ca^2+^-binding site I in hSPCA1, while site II retains its ability to bind Ca^2+^ (Fig. 2e). This may explain why hSPCA1 only transports one Ca^2+^ ion per cycle.

### A unique movement of cytosolic domains and TMD during phosphorylation

Phosphorylation is a crucial step in the transport reaction of P-type ATPases. However, hSPCA1 exhibits a distinct movement during this process compared to SERCAs and sodium-potassium pumps (Na^+^/K^+^-ATPases, NKAs).

Like SERCAs^5^ and NKAs,^44^ the cytosolic domains of hSPCA1 were loosely arranged in the CaE1 state to allow ATP binding (Supplementary information, Fig. S6e, f). In hSPCA1, the N domain interacted with the P domain through several residues, including Gly518, Ser519, Ala520, Gly521, Ile545, Glu575, Thr576, Ala579, and Ile580, with a distance between the ^480^KGA motif and the ^350^DKT motif at ~25.0 Å (Fig. 3a). Meanwhile, the A domain interacted with the P domain through multiple residues, including Val165, Gly166, Asp167, Arg168, and Leu222 in the A domain, and Val646, Asp667, Val668, and Glu671 in the P domain, with a distance of 33.2 Å from the ^350^DKT motif to the ^189^TGE motif (Fig. 3a). As expected, there was no interaction observed between the A and N domains (Fig. 3a).

The binding of AMP-PCP caused the N domain to rotate ~57.0° towards the P domain, bringing the ^480^KGA motif and the ^350^DKT motif closer together ( ~17.8 Å) (Fig. 3b). No significant conformational changes were observed in the TMD and the A and P domains during the transition of CaE1 → CaE1-ATP, with the root mean square deviation (RMSD) being 0.83 Å (Fig. 3c). In contrast, SERCA1 underwent notable movement in these domains upon ATP binding (Supplementary information, Fig. S6g), resulting in a compact headpiece with the A and N domains in extensive contact and an upward movement of TM1 (Supplementary information, Fig. S6g).^5,6^ Compared to the CaE1-ATP state in hSPCA1, the A domain and N domain of SERCA1 rotated ~18.6° and ~8.3°, respectively, towards the P domain (Fig. 3d). Moreover, TM1 moved upward by ~13.0 Å towards TM4 (Fig. 3d). Interestingly, the cytosolic domains of hSPCA1 in the CaE1-ATP state are highly similar to those of NKAs in the NaE1-ATP state,^44^ with no contact between the A and N domains (Fig. 3e).

The hydrolysis of ATP and transfer of a phosphate group to Asp350 in hSPCA1 resulted in the CaE1P-ADP state. This phosphorylation did not cause significant changes in the overall structure of hSPCA1 (RMSD is 0.87 Å) (Fig. 3c), similar to what was observed in SERCA1.^6^ In contrast, the Na^+^-bound NaE1P-ADP state of NKAs^39^ displayed a movement of the A and N domains towards each other compared to the NaE1-ATP state (Supplementary information, Fig. S6h), yielding ~15.8° and ~6.3° rotation, respectively, in comparison to the CaE1P-ADP state of hSPCA1 (Fig. 3f). This leads to a more compact headpiece and upward movement of TM1 towards TM4 ( ~11.2 Å compared to hSPCA1, Fig. 3f).

In summary, the transport process in P-type ATPases can vary within the same subfamily. The fact that the cytosolic headpiece remains open and that the TMD remains almost stationary in SPCAs during the phosphorylation reaction CaE1 → CaE1-ATP → CaE1P-ADP may represent a key feature that distinguishes them from other P-type II ATPases, such as SERCAs and NKAs.

### A compact headpiece in the Ca^2+^-occluded CaE2P state

**Fig. 4:**
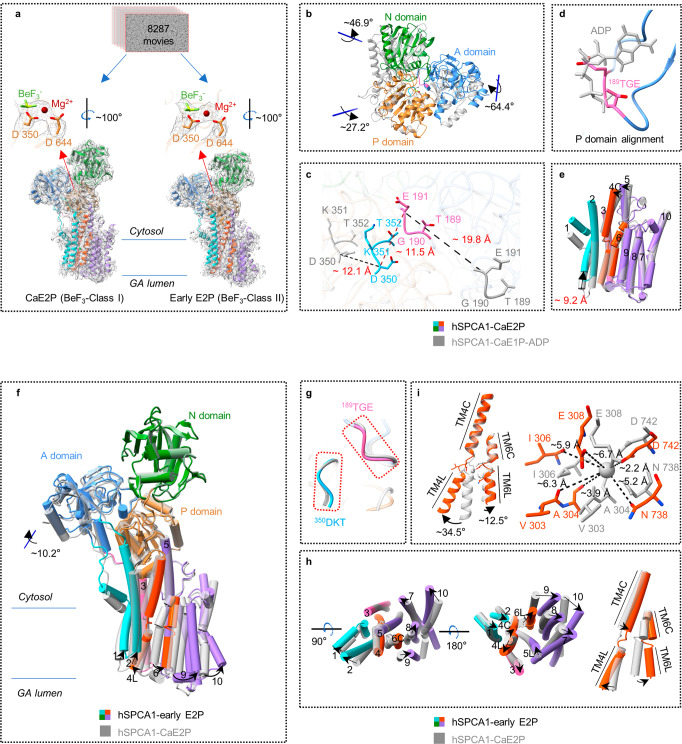
Structural details of hSPCA1 in the CaE2P and early E2P states. **a** Atomic models (color ribbon) and cryo-EM densities (dark gray mesh, contoured at 7.0 σ) of hSPCA1 in the CaE2P and early E2P states. The cryo-EM densities of BeF_3_^–^, Mg^2+^, Asp350, and Asp644 of hSPCA1 in the CaE2P and early E2P states are shown (black mesh, contoured at 10.0 σ). **b** Structural comparison of hSPCA1 cytoplasmic domains in the CaE2P (color) and CaE1P-ADP (dark gray) states by global alignment. **c** The movement of the ^189^TGE and ^350^DKT motifs from the CaE1P-ADP state to the CaE2P state. The black dashed lines show the distance between Cα of Asp350 in the CaE1P-ADP state and that in the CaE2P state, and the distance between Cα of Glu191 in the CaE1P-ADP state and that in the CaE2P state. The deep red dashed line shows the distance between Cα of Glu191 and Cα of Asp350 in the CaE2P state. **d** The conserved ^189^TGE motif (hot pink) in the CaE2P state and the ADP molecule (dark gray) in the CaE1P-ADP state. **e** Structural comparison of hSPCA1 TMD in the CaE2P (color) and CaE1P-ADP (dark gray) states. The red text shows the distance from the Cα of Leu96 in the CaE1P-ADP state to the Cα of Leu96 in the CaE2P state. **f** Structural comparison of hSPCA1 in the early E2P (color) and CaE2P (dark gray) states by global alignment. **g** Structural comparison of the ^189^TGE and ^350^DKT motifs in the early E2P (color) and CaE2P states (dark gray). **h** Structural comparison of hSPCA1 TMD in the early E2P (color) and CaE2P (dark gray) states. **i** Structural comparison of hSPCA1 TM4 and TM6 in the early E2P (orange-red) and CaE2P (dark gray) states by alignment of TM4 and TM6. The black dashed lines (right) show the distances from the Ca^2+^ ion (dark gray sphere) in the CaE2P state to the main-chain carbonyl oxygen atoms of Val303, Ala304, and Ile306, and the side-chain oxygen atoms of Glu308, Asn738, and Asp742.

To understand the movement of Ca^2+^ in the following state after the CaE1P-ADP state, we incubated the proteins with BeF_3_^–^ and 1 mM CaCl_2_ to capture the phosphorylated states of hSPCA1. This resulted in two cryo-EM maps with different conformations, BeF_3_^–^-Class I and BeF_3_^–^-Class II, with resolutions of 3.42 Å and 3.25 Å, respectively (Fig. 4a; Supplementary information, Fig. S4). The near-atomic resolutions of these two maps allowed us to trace the BeF_3_^–^ densities which were clearly visible near Asp350 (Fig. 4a).

In the BeF_3_^–^-Class I state, compared to the CaE1P-ADP state, the N domain rotated ~46.9° against the P domain, the P domain tilted ~27.2° towards the A domain, and the A domain rotated ~64.4° towards the P domain (Fig. 4b). This resulted in the movements of the conserved ^189^TGE and ^350^DKT motifs by ~19.8 Å and ~12.1 Å, respectively (Fig. 4c), shortening the distance between them ( ~11.5 Å) (Fig. 4c). The A domain extensively contacted both the P and N domains. When aligned based on the P domain, the ADP-binding site was mainly occupied by the ^189^TGE motif from the A domain (Fig. 4d), ruling out the possibility of assigning the BeF_3_^–^-Class I to the ADP-sensitive CaE1P state. Moreover, significant rotation of the cytosolic domains caused notable conformational rearrangements of TMD compared to that in CaE1P-ADP, with TM1–TM2 moving ~9.2 Å towards the cytosolic leaflet as a whole rigid body and the cytoplasmic parts of TM4 and TM5 (TM4C and TM5C) inclining towards TM1–TM2 (Fig. 4e). However, the remaining part of the TMD, including the Ca^2+^-binding residues, had a similar arrangement in both structures (RMSD is 0.74 Å; Fig. 4e; Supplementary information, Fig. S6d). Interestingly, a Ca^2+^-like density was resolved in the Ca^2+^-binding site (Supplementary information, Fig. S6d), indicating that Ca^2+^ can be stably trapped in the BeF_3_^–^-Class I state of hSPCA1; this was confirmed by MD simulation (Supplementary information, Video S1).

The structural features of an ADP-insensitive headpiece and Ca^2+^ occluded at the binding site strongly support that BeF_3_^–^-Class I should be assigned to the CaE2P state rather than the CaE1P state. Since no substrate-bound E2P structure of P-type Ca^2+^-ATPases has been reported before, our hSPCA1 structure in the CaE2P state reveals the unprecedented structural changes that occur during the CaE1P-ADP → CaE2P reaction.

### A lumen-facing cavity in the early E2P state

In the BeF_3_^–^-Class II structure, compared to the CaE2P structure, the movement of the headpiece mainly occurred in the A domain which rotated ~10.2° towards the P domain (Fig. 4f). However, the position of the ^189^TGE motif was similar in both structures (Fig. 4g), indicating that BeF_3_^–^-Class II was also trapped in an ADP-insensitive state. Interestingly, the conformations of TMDs were noticeably rearranged in BeF_3_^–^-Class II compared to CaE2P.

The rotation of the A domain in BeF_3_^–^-Class II caused TM1–TM2 to move further upwards by ~4.3 Å and TM3 by ~4.2 Å (Fig. 4f, h). The luminal part of TM4 (TM4L) swung away from TM6, whereas TM4C rotated towards it (Fig. 4h). TM5L moved towards TM6, while TM5C remained fixed. TM6–TM10 rotated away from TM4 (Fig. 4h). The separation of TM4L and TM6 created a cavity facing the GA lumen formed by TM2 and TM4–TM6. Interestingly, a large and extended EM density was observed in the lumen-facing cavity, surrounded by TM2, TM4–TM6, and TM8 (Supplementary information, Fig. S7a).

Aligning TM4 and TM6 of both BeF_3_^–^-bound structures showed no evident movement in TM4C. However, TM4L of BeF_3_^–^-Class II swung ~34.5° away from TM6L, while TM6 rotated ~12.5° away from TM4 compared to the CaE2P structure (Fig. 4i). As a result, several residues involved in Ca^2+^ binding, including Val303, Ala304, Ile306, Glu308, and Asn738, moved away from Ca^2+^ (with distances of ~6.3 Å, ~3.9 Å, ~5.9 Å, ~6.7 Å, and ~5.2 Å, respectively, Fig. 4i). This disrupted the conformation of the Ca^2+^-binding site and lowered its affinity to Ca^2+^, consistent with the absence of Ca^2+^-like EM density in the Ca^2+^-binding site of the BeF_3_^–^-Class II EM map (Supplementary information, Figs. S6d, S7a).

**Fig. 5:**
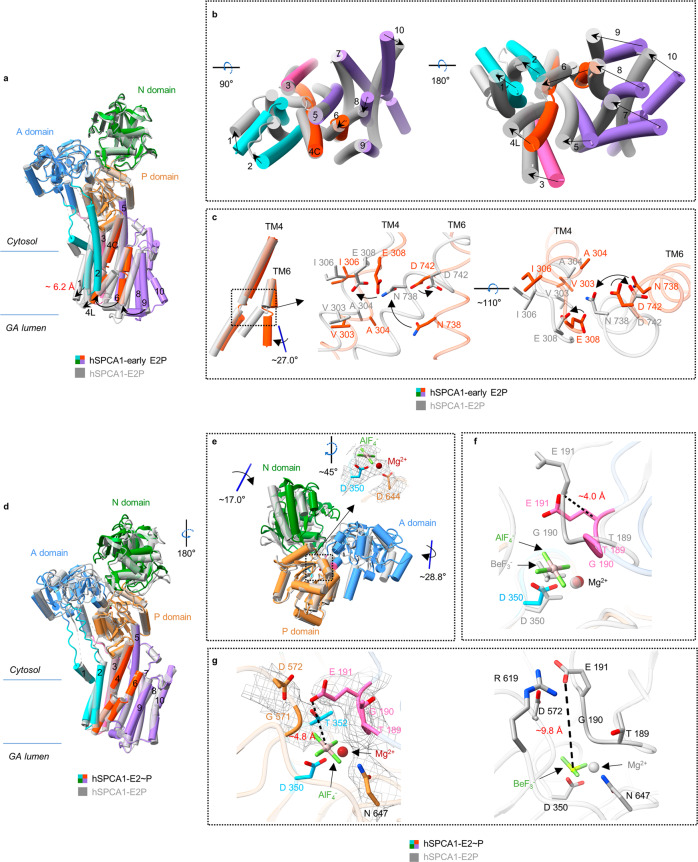
Structural comparison of hSPCA1 in three E2 states. **a** Global alignment of the early E2P (color) and E2P (dark gray, PDB: 7YAM) states of hSPCA1. The red text shows the distance from the Cα of Leu96 in the early E2P state to the Cα of Leu96 in the E2P state. **b** Structural comparison of hSPCA1 TMD in the early E2P (color) and E2P (dark gray) states. **c** Comparison of TM4 and TM6, and the Ca^2+^-binding site between the early E2P state (orange-red) and the E2P state (dark gray) in hSPCA1. **d** Global alignment of the E2~P (color) and E2P (dark gray, PDB: 7YAM) states of hSPCA1. **e** Structural comparison of hSPCA1 cytoplasmic domains in the E2~P (color) and E2P (dark gray, PDB: 7YAM) states. The upper right panel shows the cryo-EM densities of AlF_4_^–^, Mg^2+^, Asp350, and Asp644 (black mesh, contoured at 10.0 σ). **f** The movement of the ^189^TGE motif from the E2P state (dark gray, PDB: 7YAM) to the E2~P (color) state. The black dashed line show the distance between Cα of Glu191 in the E2P state and that in the E2~P state. **g** The interfaces between ^189^TGE motif and P domain in the E2~P (color) and E2P states (dark gray, PDB: 7YAM). The EM densities of the ^189^TGE motif and Thr352, Gly571, Asp572, and Asn647 in the E2~P state are shown (black mesh, contoured at 10.0 σ).

Structural comparison showed a similar headpiece (RMSD is 0.63 Å) but a different TM region in the BeF_3_^–^-Class II structure of hSPCA1 compared to the recently reported E2P structure^37^ (Fig. 5a). In the E2P structure, TM1–TM2 moved ~6.2 Å back towards the luminal leaflet of the lipid bilayer (Fig. 5a). TM3 and TM4 moved towards TM1, while TM5 bent towards TM3. Finally, the lumen-facing cavity closed due to the movement of TM6–TM10 close to TM4 (Fig. 5a, b), indicating that Ca^2+^ had been completely released from hSPCA1 in the E2P state.

It is worth noting that TM6 of the E2P state twisted ~27.0° compared to the BeF_3_^–^-Class II structure (Fig. 5c). As a result, the coordination geometry of the Ca^2+^-binding site became unsuitable for trapping Ca^2+^ (Fig. 5c). The twist of TM6 distorted the conformation of the Ca^2+^-binding site and closed the lumen-facing cavity, preventing the re-trapping of Ca^2+^.

Based on the structural features and comparisons of BeF_3_^–^-Class II to the adjacent CaE2P and E2P states, we confidently assigned BeF_3_^–^-Class II to an early E2P state of hSPCA1 along the transport cycle. The structural rearrangement during the CaE2P → early E2P → E2P transition provides clear information on Ca^2+^ release from hSPCA1.

### Conformational changes during dephosphorylation

To understand the conformational changes during hSPCA1 dephosphorylation, we trapped hSPCA1 in the E2~P state by incubating the protein with AlF_4_^–^ (Fig. 5d). The EM densities derived from AlF_4_^–^ were clearly visible near Asp350 (Fig. 5e). Superimposition of the reported E2P^37^ and our E2~P structures showed that the headpiece of hSPCA1 underwent significant rearrangements during the E2P to E2~P transition (Fig. 5d, e). Both the A and N domains moved towards the P domain, rotating ~28.8° and ~17.0°, respectively (Fig. 5e), consistent with what was observed in SERCA2b.^30^ This changed the interface between the ^189^TGE motif and the P domain during the E2P to E2~P transition (Fig. 5f, g). In the E2P state, Asp572, Arg619, and Asn647 were essential for the interaction of the ^189^TGE motif with the P domain (Fig. 5g). However, in the E2~P structure, the ^189^TGE motif interacted with Thr352, Gly571, Asp572, and Asn647 in the P domain, moving Glu191 closer to the phosphorylated Asp350 (Fig. 5f, g), which is important for initiating dephosphorylation.^45,46^

As domain A rotated, TMD experienced a slight shift, with the lumen-facing cavity remaining closed (Fig. 5d), suggesting that the dephosphorylation process has minimal effect on the gating of the Ca^2+^ release pathway.

### A full Ca^2+^ transport pathway

To better understand the complete Ca^2+^ transport pathway, we used explicit solvent all-atom MD simulations to study the four key states (CaE1, CaE2P, early E2P, and E2~P). These simulations allowed us to study the hydration of the substrate-binding site and solvent-accessible pathways in detail, which are challenging to capture in the cryo-EM structures.

**Fig. 6:**
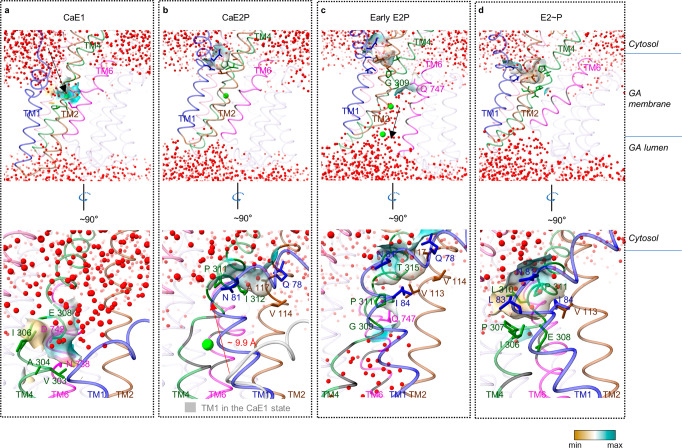
The Ca^2+^ transport pathway of hSPCA1. **a** The open cytosol-facing cavity of hSPCA1 in the CaE1 state. TM1, TM2, TM4, and TM6 are colored medium blue, saddle brown, forest green, and magenta, respectively. Water molecules and Ca^2+^ are shown as small red balls and the lime sphere, respectively. The black arrow indicates the possible Ca^2+^ entry pathway. **b** The closed cytosol-facing cavity of hSPCA1 in the CaE2P state. The red arrow (bottom) shows the distance from the Cα of Pro82 in the CaE1 state to the Cα of Pro82 in the CaE2P state. **c** The closed cytosol-facing cavity and open lumen-facing cavity of hSPCA1 in the early E2P state. The black arrow indicates the Ca^2+^ release pathway. **d** The semi-open cytosol-facing cavity and closed lumen-facing cavity of hSPCA1 in the E2~P state. The Ca^2+^-binding residues are colored forest green, magenta, or black. The surfaces are shown with colors ranging from dark cyan (most hydrophilic) to white (intermediate) to dark goldenrod (most lipophilic).

MD simulations of the CaE1 state showed a solvent-accessible cavity facing the cytosol formed by TM1, TM2, and TM4 (Fig. 6a). This cavity provided access to the Ca^2+^-binding site, suggesting a possible entry pathway for Ca^2+^. It remained open during the transition of CaE1 → CaE1-ATP → CaE1P-ADP. In the CaE2P state, the Ca^2+^-binding site was still intact, but the kink in TM1 moved towards the cytoplasmic side. As a result, the cavity was blocked by several amino acids, including Gln78, Asn81, Val114, Ala117, Pro311, and Ile312, making the cavity dehydrated and sealing the Ca^2+^ within the TMD (Fig. 6b; Supplementary information, Fig. S6d and Video S1).

In the following early E2P state, TM4L and TM6 moved apart (Fig. 4h, i), creating a highly hydrated cavity facing the lumen (Fig. 6c; Supplementary information, Video S2). This suggests a potential pathway for releasing Ca^2+^. The lumen-facing cavity was blocked in the middle of the membrane by two amino acids, Gly309 and Gln747, while the cytosol-facing cavity was closed at the membrane surface. These separate cavities prevented Ca^2+^ backflow and allowed unidirectionally transport from the cytosol to the GA lumen (Fig. 6c).

In the subsequent E2~P state, MD simulations showed that the lumen-facing cavity became dehydrated, indicating closure of the Ca^2+^ release pathway (Fig. 6d). At the same time, movement of TM1–TM2 towards the luminal side partially opened the cytosol-facing cavity (Fig. 6d). This allowed solvent from the cytosol to reach the surface above the Ca^2+^-binding site (Fig. 6d). Since the TMD conformation of the recently reported E2P state is almost identical to our E2~P structure (Fig. 5d), it is likely that the partially opened cytosol-facing cavity is already formed in the previous E2P state.

By cryo-EM analysis of hSPCA1 and all-atom MD simulations, we were able to deduce a complete Ca^2+^ transport pathway for hSPCA1 based on changes in hydration and openness of the cytosol-facing and lumen-facing cavities.

### A proposed model of Ca^2+^ transport by hSPCA1

**Fig. 7:**
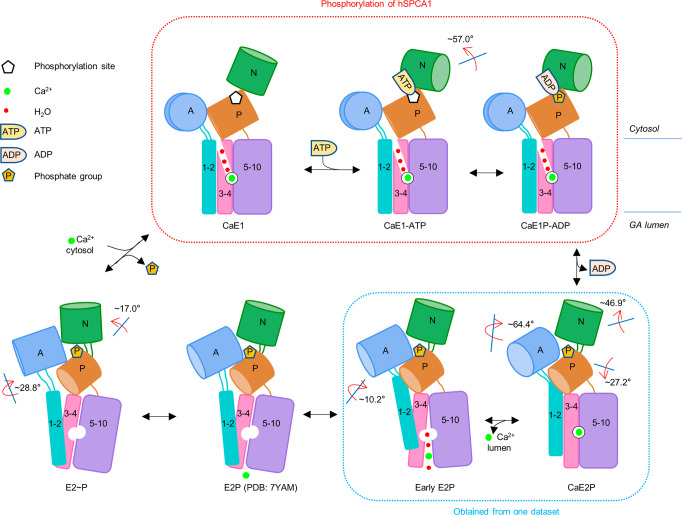
A proposed model of Ca^2+^ transport by hSPCA1. Schematic models of the stages of the transport cycle in hSPCA1. A, N, and P domains are shown as light cornflower blue, light forest green, and peru cylinders, respectively. TM1–TM2, TM3–TM4, and TM5–TM10 are shown as cyan, light pink, and light-medium purple rectangles. The red arrows indicate movements of the cytosolic domains (with respect to the previous state in the forward reaction).

Based on all our solved cryo-EM structures and MD simulation results of hSPCA1, as well as the reported E2P structure,^37^ we analyzed the structural basis and rearrangement for Ca^2+^ binding, translocation and release, as well as the Ca^2+^ entry and releasing pathway of hSPCA1. This allows us to propose a model of how hSPCA1 transports Ca^2+^ across the GA membrane (Fig. 7; Supplementary information, Video S3).

During the Ca^2+^ transport cycle, hSPCA1 starts with a loosely arranged headpiece of cytosolic domains, with the N domain ready to bind ATP. Ca^2+^ from the cytosol can then enter the binding site through the cytosol-facing cavity. The N domain binds an ATP molecule and rotates ~57.0°, followed by ATP hydrolysis and phosphorylation of Asp350. This leads to the phosphorylated E1 state of hSPCA1 (the CaE1P-ADP state) with little changes in the overall structure. After ADP release, but prior to Ca^2+^ release, the A and N domains rotate towards and against the P domain, respectively, forming a compact headpiece. The conserved ^189^TGE motif moves close to the phosphorylation site Asp350 (the CaE2P state; Figs. 4c, 7a). As a result, the integrated movement of TM1–TM2 closes the cytosol-facing cavity and dehydrates the Ca^2+^-binding site, resulting in a Ca^2+^-occluded CaE2P state. During the transition from the CaE2P state to the early E2P state, TM4L and TM6 move apart to disrupt the Ca^2+^-binding site and create a large lumen-facing cavity formed by TM2, TM4–TM6 for Ca^2+^ release. Further movement of TM4 and TM6 in the E2P state closes the lumen-facing cavity, ensuring the complete release of Ca^2+^. The movement of TM1–TM2 towards the luminal leaflet partially opens the cytosol-facing cavity. After entering the E2~P state, the headpiece rearranges for dephosphorylation, the A and N domains move towards the P domain, whereas the P domain and TMD remain unchanged. Finally, dephosphorylation of Asp350 loosens the compact headpiece, and makes TM1–TM2 move back towards the luminal leaflet. A new Ca^2+^ from the cytosol enters the binding site to begin a new transport cycle.

## Discussion

In our study, we determined six cryo-EM structures of hSPCA1 representing the CaE1, CaE1-ATP, CaE1P-ADP, CaE2P, early E2P, and E2~P states, respectively. Together with the previously reported E2P state, these results provide new insights into the dynamics of hSPCA1 during the phosphorylation and dephosphorylation process involved in Ca^2+^ transport.

### Structures of hSPCA1 in the CaE1-ATP state bearing different conformations

Recently, Chen et al.^37^ reported a CaE1-ATP structure that showed a compact headpiece and upward movement of TM1 and TM2 towards the cytoplasm compared to our CaE1-ATP structure (Supplementary information, Fig. S7c). The conformational differences between the reported and our structures may be due to different sample preparation methods. In the reported CaE1-ATP structure, a megabody 14 (Mb14) with a molecular weight of ~56 kDa was used to stabilize the conformation of hSPCA1 while biochemically abolishing its Ca^2+^-dependent ATPase activity.^37^ The binding of Mb14, with its relatively large volume, to the headpiece would limit hSPCA1’s conformational rearrangements. In contrast, we prepared our cryo-EM sample of the CaE1-ATP-state hSPCA1 by directly incubating the ATP analog with hSPCA1 protein, a method which was widely used to trap the E1-ATP state of the representative P-type ATPases.^6,39,47,48^

### TGE motif moves towards the phosphorylation site before Ca^2+^ release

The TGE motif, which is believed to be closely related to protein dephosphorylation,^33,42,45,46^ undergoes a large movement during substrate transport. However, the coupling of this movement with substrate transport is not well understood. A recent study showed that in the CaE1P state of hSERCA2, the TGE motif was located in a similar position to that in the CaE1-ATP structure, far from the phosphorylated aspartic acid ( ~27.0 Å; Supplementary information, Fig. S7d).^30^ In our study, we determined the CaE2P structure, providing new insights into the movement of the TGE motif. Compared to the reported CaE1P structure of hSERCA2 (Supplementary information, Fig. S7d), our CaE2P structure of hSPCA1 shows that the TGE motif is positioned near the phosphorylation site prior to Ca^2+^ release. We speculate that the movement of the TGE motif likely occurs during the transition from the CaE1P state to the CaE2P state, and that Ca^2+^ release may be triggered only in the subsequent transition.

### Ca^2+^ release triggered by a ~10.2° rotation of the A domain

It is generally accepted that the rotation of the A domain is coupled with Ca^2+^ release,^33,49–51^ but the detailed structural mechanism remains unclear. Our structures revealed that during the transition from the CaE2P to early E2P states, the cytosolic domains maintain almost identical conformations except for a ~10.2° rotation of the A domain (Fig. 4f). This rotation induces a series of conformational changes in the TMD (Fig. 4h), including the upward movement of TM1–TM2 towards the cytosolic leaflet, TM3 moving away from TM1, and TM4L moving away from TM6. Additionally, TM6–TM10 move away from TM4, resulting in the destruction of the Ca^2+^-binding site and the formation of the lumen-facing cavity that allows Ca^2+^ to be released into the GA lumen. Therefore, the small rotation of the A domain during the transition from CaE2P to early E2P state may play a crucial role in inducing Ca^2+^ release.

### A putative regulator of hSPCA1

Previous studies have shown that small-molecule drugs targeting the lumen-facing cavity formed by TM2 and TM4–TM6 in NKAs are effective in treating heart diseases.^52^ In the early E2P structure of hSPCA1, we also observed a blob of EM density in the lumen-facing cavity, surrounded by Asp102 in TM2, Ala304 and Glu308 in TM4, Ile717 in TM5, Asn738, Asp742, and Gln747 in TM6, and Thr811 in TM8 (Supplementary information, Fig. S7a, b). Our MD simulations suggest that this cavity is highly hydrated (Fig. 6c). Therefore, we hypothesize that the unassigned EM density in the cavity may be derived from certain cations and/or hydrophilic molecules that could potentially regulate the transport activity of hSPCA1. Further study is needed to confirm this hypothesis.

In summary, by determining the structures of six consecutive intermediate states along the Post-Albers reaction cycle, we were able to define a near-complete Ca^2+^ transport pathway for hSPCA1. The specific Ca^2+^ transport mechanism of hSPCA1 differs from that of SERCAs and other P-type II ATPases in many ways. This information not only expands our understanding of P-type ATPases, but also holds great potential for the development of specific agonists and inhibitors targeting hSPCA1, which could be beneficial in treating diseases related to hSPCA1.

## Materials and methods

### DNA constructs

The full-length hSPCA1a-coding sequence (NM_001199179.1) was obtained by PCR amplification from HEK293T cDNA. The coding sequence was cloned into the pCAG vector and fused with a C-terminal 2× Strep tag.

### Protein expression and purification

HEK293F cells were cultured in SMM 293-TII medium (Sino Biological Inc.) supplemented with 1× penicillin/streptomycin at 37 °C with 5% CO_2_. Each liter of cells was transiently transfected with 1.8 mg DNA using 4.5 mg polyethyleneimine (Polysciences, Inc.) at a density of 2.0 × 10^6^ to 2.5 × 10^6^ cells/mL. The transfected cells were cultured for 48 h before being harvested. About 4 L of transfected cells were used for one batch of protein purification. All procedures below were carried out at 4 °C or on ice.

The cell pellet was resuspended and solubilized in lysis buffer containing 100 mM Tris (pH 8.0), 100 mM KCl, 5 mM MgCl_2_, 1 mM CaCl_2_, 20% (v/v) glycerol, 1% (w/v) DDM (Anatrace) & 0.2% (w/v) CHS (Sigma), 1 mM DTT, 1 mM PMSF, and 1× EDTA-free protease inhibitor cocktail (Roche), and stirred gently for 2 h. After centrifugation at 20,000× *g* for 1 h, the supernatant was collected and incubated with Strep-Tactin® Sepharose® (IBA, LifeSciences) for 1.5 h. The resin was washed with 50 column volumes of wash buffer containing 100 mM Tris (pH 8.0), 100 mM KCl, 5 mM MgCl_2_, 1 mM CaCl_2_, 10% (v/v) glycerol, 0.05% (w/v) DDM & 0.01% (w/v) CHS, 1 mM DTT, and 1 mM PMSF. Subsequently, the protein was eluted with 50 mM Tris (pH 8.0), 100 mM KCl, 5 mM MgCl_2_, 1 mM CaCl_2_, 0.025% (w/v) DDM & 0.005% (w/v) CHS and 50 mM biotin (VETEC). The protein was concentrated using a 50-kDa MWCO Amicon Ultra centrifugal filter. The protein was further purified by SEC using a Superose 6 Increase 5/150 GL column (GE Healthcare) equilibrated with 25 mM Tris (pH 8.0), 100 mM KCl, 5 mM MgCl_2_, 1 mM CaCl_2_, and 0.06% (w/v) digitonin (Sigma). 1 mM CaCl_2_ was absent in the SEC buffer for the E2~P state. Peak fractions were collected for Cryo-EM sample preparation and ATPase activity assay.

### ATPase activity assay

The peak fractions after SEC were diluted to a suitable concentration and used in an ATPase activity assay. The assay was performed using a commercially available kit by measuring the release of inorganic phosphate (Pi) from ATP according to the kit protocol (Nanjing Jiancheng Bioengineering Institute, China). The absorbance was measured at 636 nm using a microplate reader (Tecan, Switzerland). The protein concentration was determined by Implen P330 ultra-micro spectrophotometer.

### Preparation of Cryo-EM samples

Biochemical experiments and structural data have proven that the ligation of BeF_3_^–^ at the aspartic acid phosphorylation site would simulate the CaE1P, CaE2P, and E2P states.^29–31,38^ AlF_4_^–^ possesses planar geometry, which coordinates the Asp-oxygen and the hydrolytic water at apical positions, producing the bipyramidal structure superimposable to the penta-coordinated phosphorus in the transition state (E2~P) during the aspartylphosphate hydrolysis.^29,38,53^ Thus, the purified hSPCA1 (concentrated to ~10 mg/mL) was incubated with different substrates to trap different intermediate states along the transport cycle using the following conditions: the CaE1-ATP state was captured with 1 mM ATP analog β,γ-methyleneadenosine 5’-triphosphate (AMP-PCP); the CaE1P-ADP state was captured with 5 mM ADP, 5 mM NaF, and 1 mM AlCl_3_; the CaE2P/early E2P states were captured with 10 mM NaF and 2 mM BeSO_4_; and the E2~P state was captured with 10 mM NaF and 2 mM AlCl_3_. After incubating with substrates for 1 h on ice, the protein solutions were applied to a freshly glow-discharged Quantifoil holey carbon grid (PELCO easiGlow, 0.39 mBar, air, 15 mA, 40 s, R1.2/1.3, Au, 300 mesh). The cryo-EM samples were prepared using a Vitrobot Mark IV (Thermo Fischer Scientific) at 4 °C with a blotting time of 3–4 s under 99% humidity conditions, and then the grids were plunge-frozen in liquid ethane. All grids were then transferred to liquid nitrogen and stored there for data collection. For the CaE1 state, hSPCA1 samples were freshly purified and concentrated in SEC buffer, then used immediately.

### Cryo-EM data collection

Cryo-EM data were collected by EPU2 software package (Thermo Fisher Scientific) on a 300 kV Titan Krios G3i transmission electron microscope (Thermo Fischer Scientific). Prior to detection, inelastically scattered electrons were filtered out with a GIF Quantum energy filter (Gatan) using a slit width of 20 eV. Images were acquired in counting mode (super resolution) on a K3 Summit detector (Gatan) at a nominal magnification of 105,000×, resulting in a pixel size of 0.84 Å/pixel. Images were exposed for a total of 1.83 s with a dose rate of 20 e^–^/pixel/s resulting in a total dose of 50 e^–^/Å^2^, which was fractionated into 32 frames.

### Cryo-EM data processing

Movie frames were aligned using the Patch motion correction (multi) integrated in cryoSPARC (v3.3). The contrast transfer function (CTF) parameters were estimated from the aligned micrographs using Patch CTF estimation (multi) in cryoSPARC. One thousand images were used to generate an initial particle-set by blob picker in cryoSPARC; particle extraction was carried out with a box size of 360 pixels; and two-dimensional (2D) classification was performed in cryoSPARC. High-quality 2D class averages representing projections in different orientations were selected as templates for Topaz training of the entire dataset. The particles were then subjected to 2D classification in cryoSPARC or 3D classification in Relion. After ab-initio model building and heterogeneous refinement in cryoSPARC, most bad particles were removed, and the best class was used to generate the final map by using Non-uniform refinement in cryoSPARC. Subtraction of detergent density and local refinement yielded an improved map with better details using cryoSPARC (v3.3). The resolution was estimated by using the gold-standard FSC 0.143 criterion.

### Model building and refinement

The model of the hSPCA1 was built by fitting the model predicted by AlphaFold2^54^ into the density map using UCSF ChimeraX,^55^ followed by manual model construction in COOT^56^ and real-space refinement with secondary structure restraints in PHENIX.^57^ The model statistics are presented in Supplementary information, Table S1.

### MD simulations

The models of hSPCA1 in four different states (CaE1, CaE2P, early E2P, and E2~P) were constructed using the corresponding cryo-EM structures. These models were then embedded into a flat, mixed lipid bilayer consisting of 1-palmitoyl-2-oleoyl-sn-glycero-3-phosphocholine (POPC), and solvated in a cubic water box containing 0.10 M KCl and 0.1 M CaCl_2_. The size of the box was 12.6 nm, 12.6 nm, and 12.9 nm in the *x*, *y*, and *z* dimensions, respectively, resulting in ∼210,000 atoms in total for each model. The Orientations of Proteins in Membranes (OPM) webserver was used to align the TMD of hSPCA1 in the lipid bilayer. The systems were built with the CHARMM-GUI webserver^58^ and underwent an energy minimzation step using the steepest descent algorithm followed by a six-step equilibration during which position constraints in the systems were gradually removed. Finally, production runs in semi-iso-thermal-isobaric (NPT) conditions were performed. The CHARMM36m force field^59^ was used for proteins, CHARMM36 for lipids, and TIP3P for water. Force field parameters used for the phosphorylated Asp350 were the same as previously described.^60^

In the MD simulations, the temperature was kept constant at 310 K using a Nose–Hoover thermostat with a 1 ps coupling constant, and the pressure was kept at 1.0 bar using the Parrinello–Rahman barostat with a 5 ps coupling constant. A cut-off of 1.2 nm was applied for the van der Waals interactions using a switch function starting at 1.0 nm. The cut-off for the short-range electrostatic interactions was also set at 1.2 nm and the long-range electrostatic interactions were calculated by means of the particle mesh Ewald decomposition algorithm with a mesh spacing of 0.12 nm. A reciprocal grid of 108 × 108 × 108 cells was used with fourth-order B-spline interpolation. All simulations were performed using a GPU-accelerated version of Gromacs 2021.5.^61^ 500 ns or 1000 ns simulation was performed for each model. Trajectories were analyzed using PLUMED.^62^ Averaged density maps of the water molecules were analyzed using GROMAPs.^63^

## Supplementary information

Supplementary information, Fig. S1

Supplementary information, Fig. S2

Supplementary information, Fig. S3

Supplementary information, Fig. S4

Supplementary information, Fig. S5

Supplementary information, Fig. S6

Supplementary information, Fig. S7

Supplementary information, Table S1

Supplementary information, Video S1

Supplementary information, Video S2

Supplementary information, Video S3

Supplementary information, Video legend

## Data Availability

The cryo-EM structures of hSPCA1 in CaE1, CaE1-ATP, CaE1P-ADP, CaE2P, early E2P, and E2~P states have been deposited at the Protein Data Bank (PDB) with the accession codes 8IWP, 8IWR, 8IWW, 8IWS, 8IWT and 8IWU, respectively. The cryo-EM density maps of these structures have been deposited at the Electron Microscopy Data Bank (EMDB) with the codes EMD-35776, EMD-35777, EMD-35781, EMD-35778, EMD-35779, and EMD-35780, respectively.
