# Supplementary material for: Structure and transport mechanism of the human calcium pump SPCA1
Source: Cell Res. 2023 May 31;33(7):533–45. doi: 10.1038/s41422-023-00827-x (PMC10313705; doi:10.1038/s41422-023-00827-x)
Supplement: Supplementary file 1 — Supplementary information, Fig. S1 [file 41422_2023_827_MOESM1_ESM.pdf]

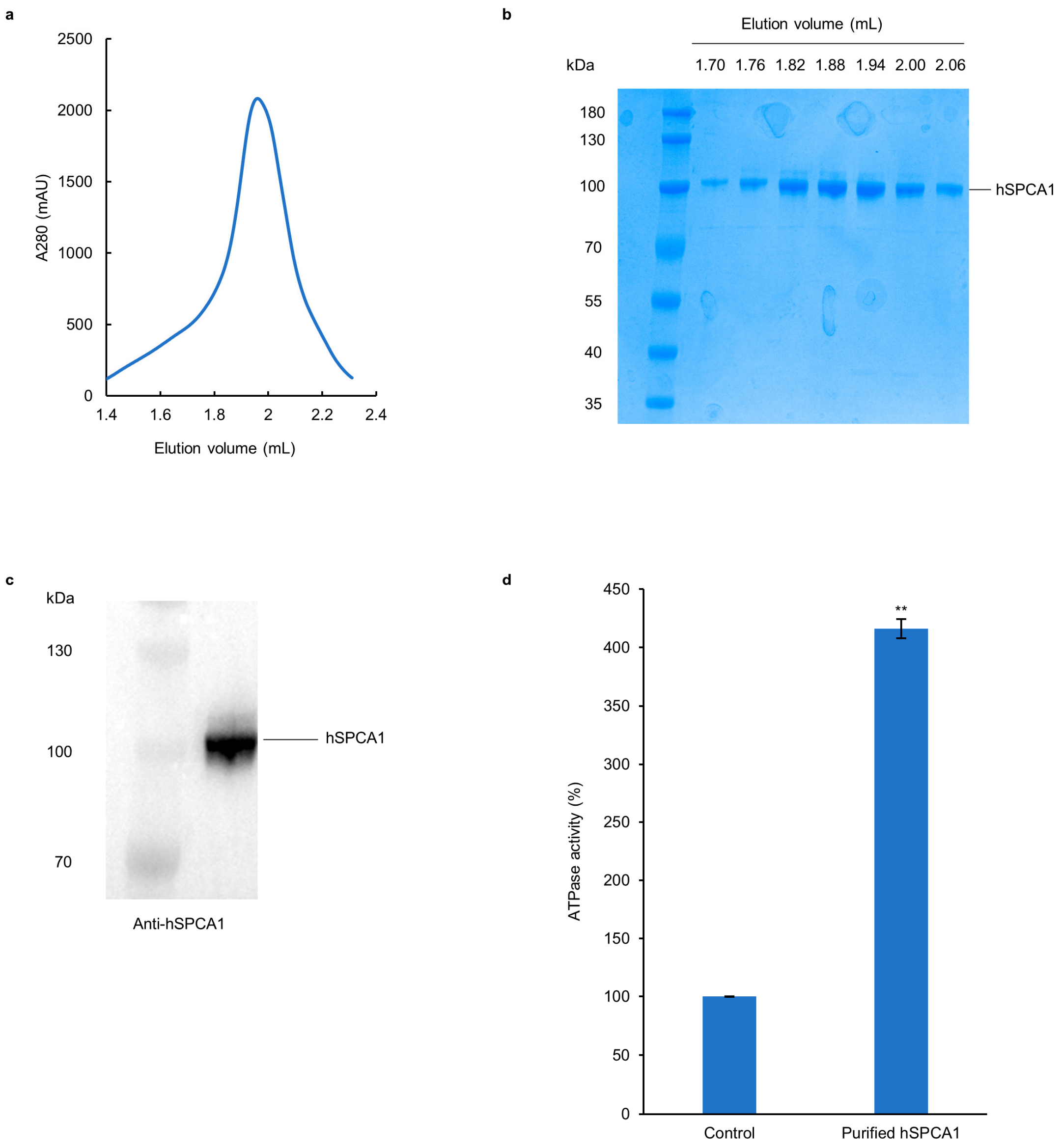

Supplementary information, Fig. S1. Biochemical analysis of hSPCA1. a, Size-exclusion chromatography profile of hSPCA1. **b,** SDS-PAGE analysis of purified hSPCA1 from the SEC peak fractions. **c,** Western blot analysis of the purified hSPCA1. Immunostaining was carried out with a hSPCA1-specific polyclonal antibody (1:1000 dilution, Abclonal: A2515). **d,** ATPase activity assay of purified hSPCA1. Data are presented as the mean ± SE of three independent measurements after normalization. Statistical significance compared with the control is shown: **$p < 0.01$.
