# Supplementary material for: Structure and transport mechanism of the human calcium pump SPCA1
Source: Cell Res. 2023 May 31;33(7):533–45. doi: 10.1038/s41422-023-00827-x (PMC10313705; doi:10.1038/s41422-023-00827-x)
Supplement: Supplementary file 2 — Supplementary information, Fig. S2 [file 41422_2023_827_MOESM2_ESM.pdf]

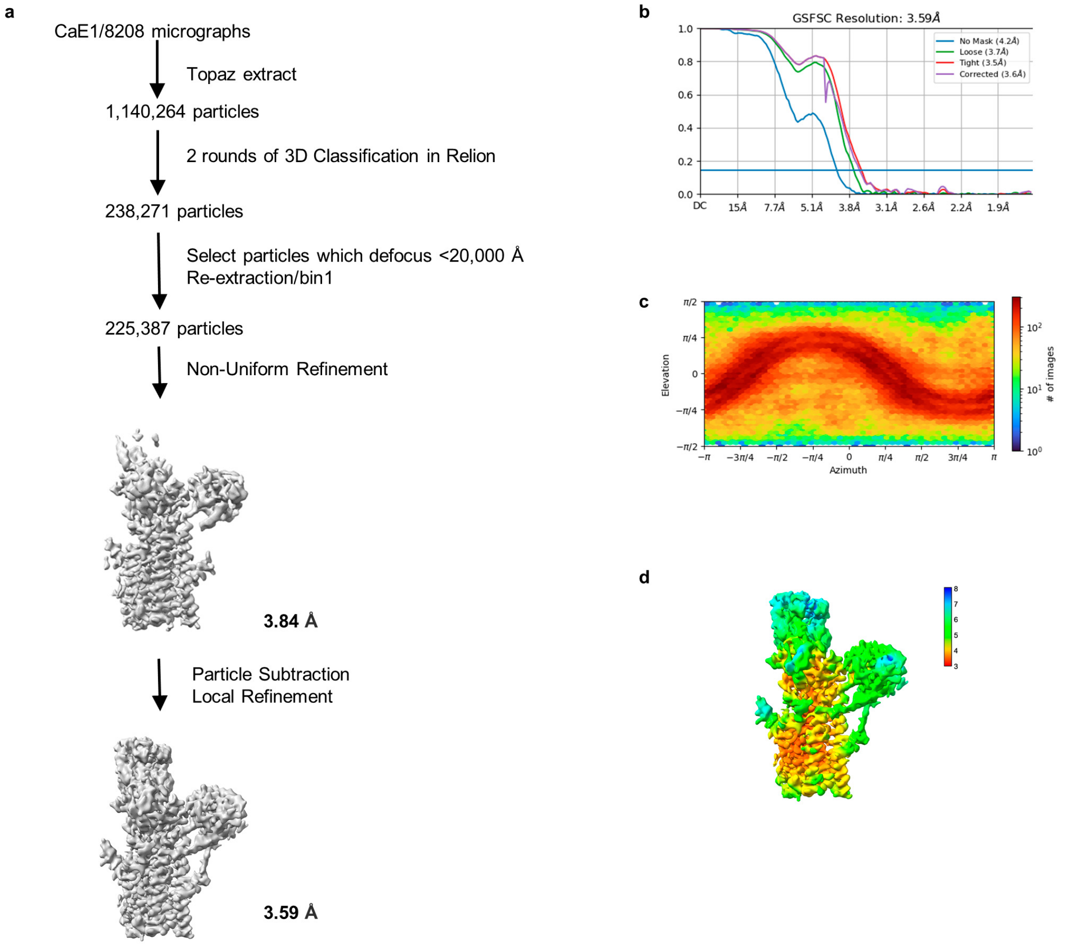

Supplementary information, Fig. S2. Cryo-EM analysis of hSPCA1 in the CaE1 state. a, Processing workflows of cryo-EM data in the CaE1 state. **b,** Gold-standard Fourier Shell correlation (FSC=0.143) curve of hSPCA1 in the CaE1 state after 3D refinement. **c,** Particle orientation distributions in the last iteration of the structural refinement of hSPCA1 in the CaE1 state. **d,** Local resolution estimation of the final 3D density map of hSPCA1 in the CaE1 state.
