# Supplementary material for: Structure and transport mechanism of the human calcium pump SPCA1
Source: Cell Res. 2023 May 31;33(7):533–45. doi: 10.1038/s41422-023-00827-x (PMC10313705; doi:10.1038/s41422-023-00827-x)
Supplement: Supplementary file 3 — Supplementary information, Fig. S3 [file 41422_2023_827_MOESM3_ESM.pdf]

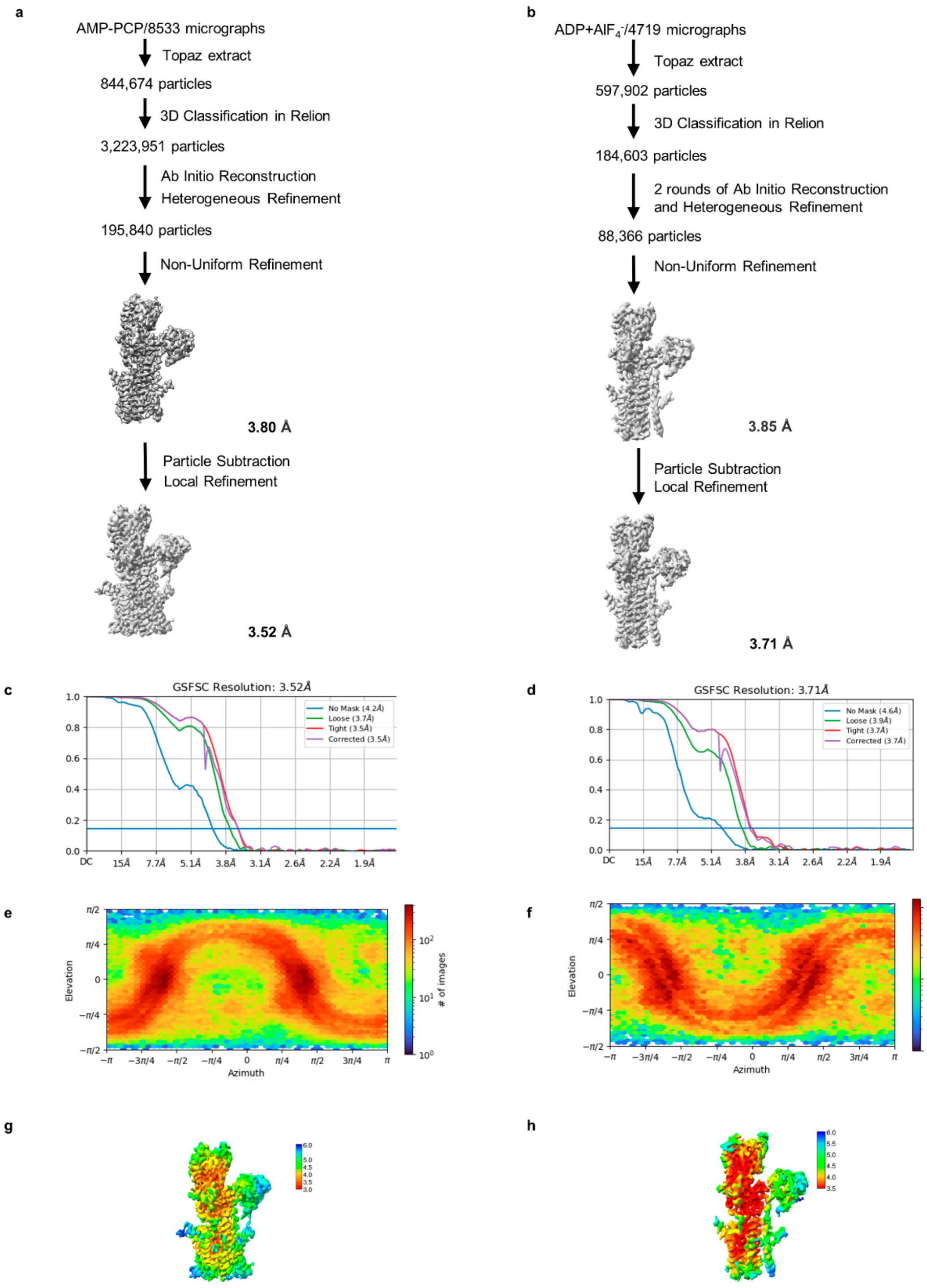

Supplementary information, Fig. S3. Cryo-EM analysis of hSPCA1 in the CaE1-ATP state and the CaE1P-ADP state. a, Processing workflows of Cryo-EM data in the CaE1-ATP state. **b,** Processing workflows of Cryo-EM data in

the CaE1P-ADP state. **c, d,** Gold-standard Fourier Shell correlation (FSC=0.143) curves of hSPCA1 in the CaE1-ATP state (**c**) and the CaE1P-ADP state (**d**) after 3D refinement. **e, f,** Particle orientation distributions in the last iteration of the structural refinement of hSPCA1 in the CaE1-ATP state (**e**) and the CaE1P-ADP state (**f**). **g, h,** Local resolution estimation of the final 3D density map of hSPCA1 in the CaE1-ATP state (**g**) and the CaE1P-ADP state (**h**).
