# Supplementary material for: Structure and transport mechanism of the human calcium pump SPCA1
Source: Cell Res. 2023 May 31;33(7):533–45. doi: 10.1038/s41422-023-00827-x (PMC10313705; doi:10.1038/s41422-023-00827-x)
Supplement: Supplementary file 4 — Supplementary information, Fig. S4 [file 41422_2023_827_MOESM4_ESM.pdf]

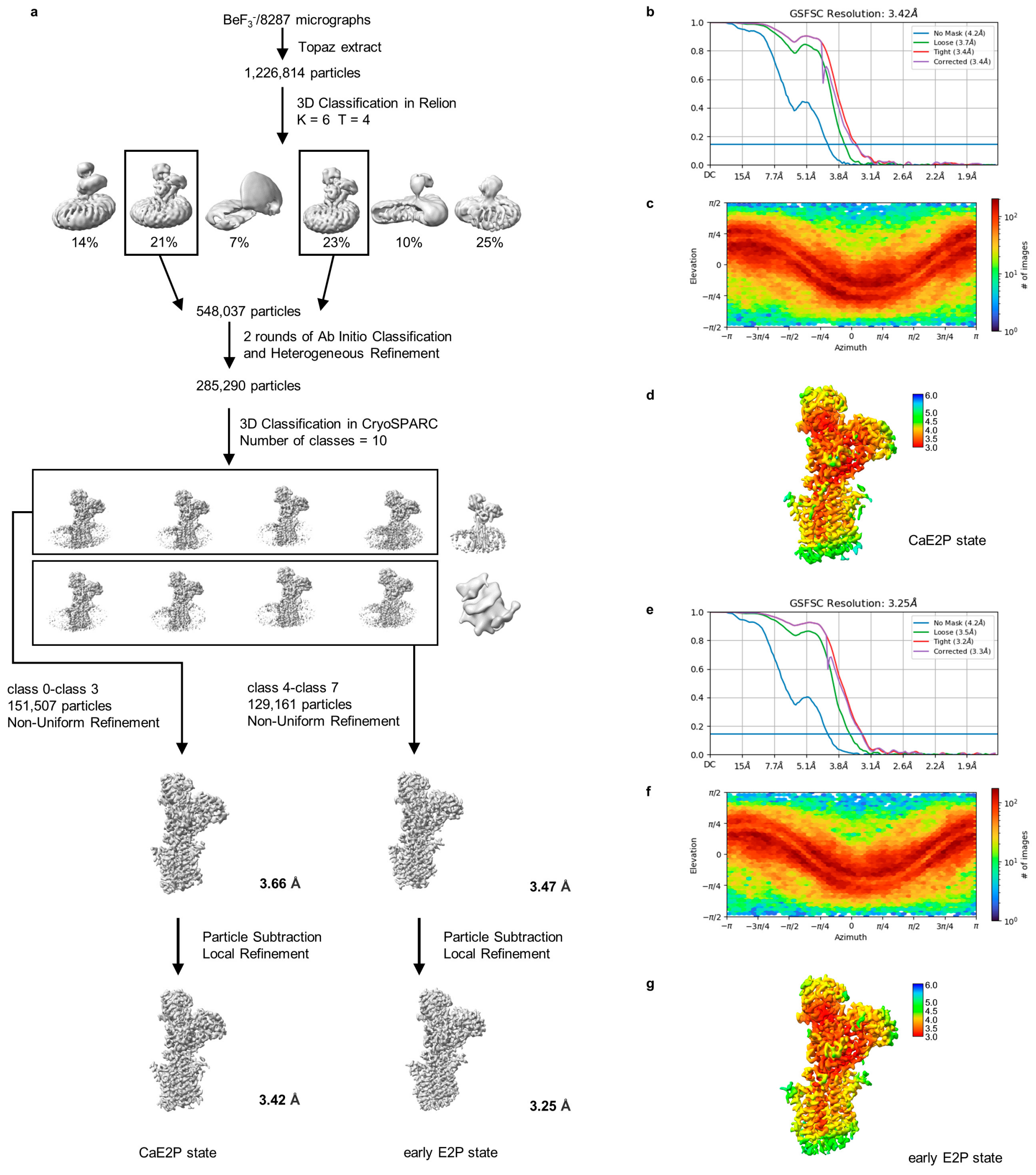

Supplementary information, Fig. S4. Cryo-EM analysis of hSPCA1 in the CaE2P and early E2P states. a, Processing workflows of Cryo-EM data in the CaE2P and early E2P states. **b,** Gold-standard Fourier Shell correlation (FSC=0.143) curves of hSPCA1 in the CaE2P state after 3D refinement. **c,** Particle orientation distributions in the last iteration of the structural refinement of hSPCA1 in the CaE2P state. **d,** Local resolution estimation of the final 3D density map of hSPCA1 in the CaE2P state. **e,** Gold-standard Fourier Shell

correlation (FSC=0.143) curves of hSPCA1 in the early E2P state after 3D refinement. **f,** Particle orientation distributions in the last iteration of the structural refinement of hSPCA1 in the early E2P state. **g,** Local resolution estimation of the final 3D density map of hSPCA1 in the early E2P state.
