# Supplementary material for: Structure and transport mechanism of the human calcium pump SPCA1
Source: Cell Res. 2023 May 31;33(7):533–45. doi: 10.1038/s41422-023-00827-x (PMC10313705; doi:10.1038/s41422-023-00827-x)
Supplement: Supplementary file 6 — Supplementary information, Fig. S6 [file 41422_2023_827_MOESM6_ESM.pdf]

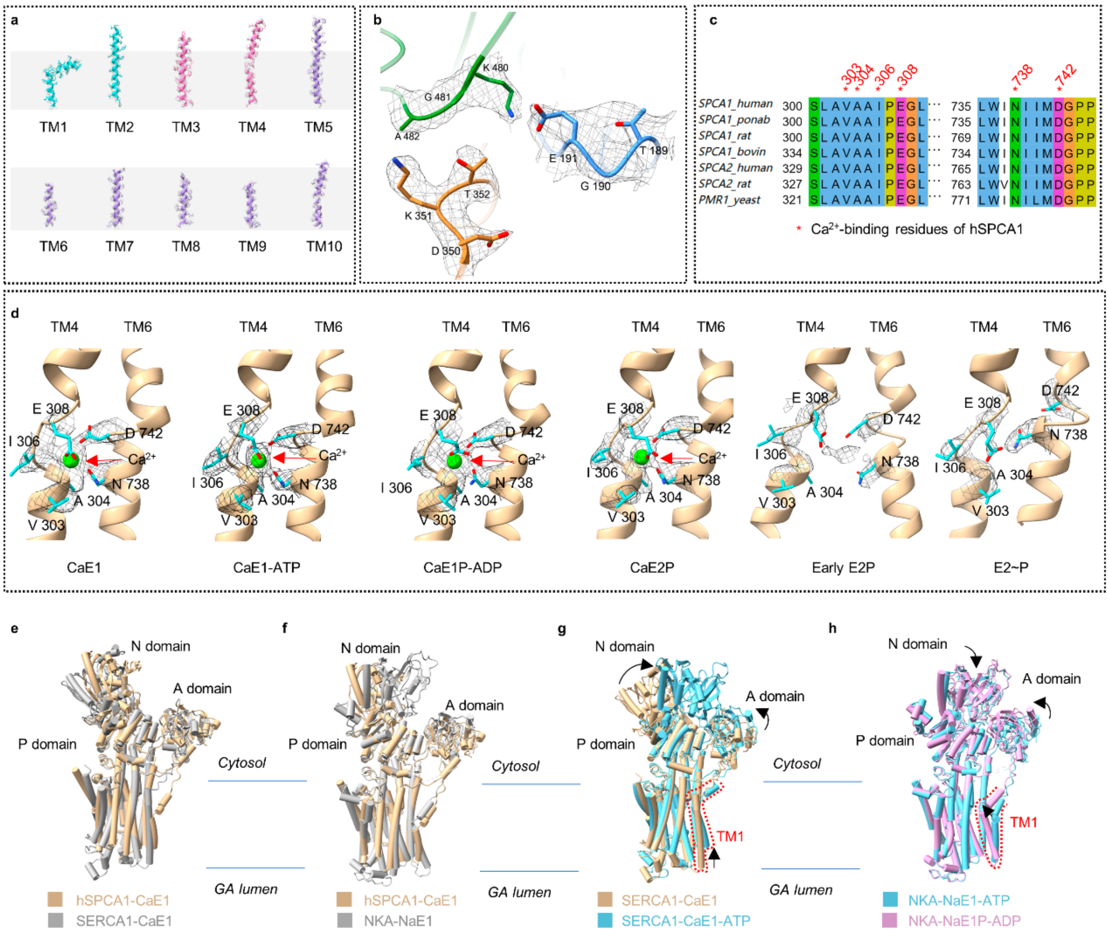

Supplementary information, Fig. S6. Structural and conservative analysis of hSPCA1. a, TM helices and cryo-EM densities (dark gray mesh, contoured at 10.0 sigma) of hSPCA1 in the CaE1-ATP state. **b,** Cryo-EM maps and ribbon models of three conserved motifs of hSPCA1 in the CaE1-ATP state: ^{480}KGA in the N domain (contoured at 9.3 sigma), ^{350}DKT in the P domain (contoured at 8.8 sigma), and ^{189}TGE in the A domain (contoured at 6.0 sigma). **c,** Sequence alignment of the Ca^{2+}-binding residues of hSPCA1 and its homologs in human and other species. **d,** Cryo-EM densities of Ca^{2+} and Ca^{2+}-binding residues in six different states of hSPCA1. Cryo-EM maps of Ca^{2+} plus Ca^{2+}-binding sites are shown as black mesh (contoured at 13.0 sigma). Ca^{2+} and Ca^{2+}-binding residues are shown as lime sphere and cyan sticks, respectively. **e,** Structural comparison of hSPCA1 in the CaE1 state (tan) and SERCA1 in the CaE1 state

(dark gray, PDB: 1SU4). **f,** Structural comparison of hSPCA1 in the CaE1 state (tan) and the α-subunit of NKA in the Na-bound NaE1 state (dark gray, PDB: 7E1Z). **g.** Structural comparison of SERCA1 in the CaE1 state (tan, PDB: 1SU4) and in the CaE1-ATP state (light sky blue, PDB:1T5S). **h,** Structural comparison of the α-subunit of NKA in the NaE1-ATP state (light sky blue, PDB: 8D3W) and in the NaE1P-ADP state (plum, PDB: 8D3U).
