# Supplementary material for: Structure and transport mechanism of the human calcium pump SPCA1
Source: Cell Res. 2023 May 31;33(7):533–45. doi: 10.1038/s41422-023-00827-x (PMC10313705; doi:10.1038/s41422-023-00827-x)
Supplement: Supplementary file 7 — Supplementary information, Fig. S7 [file 41422_2023_827_MOESM7_ESM.pdf]

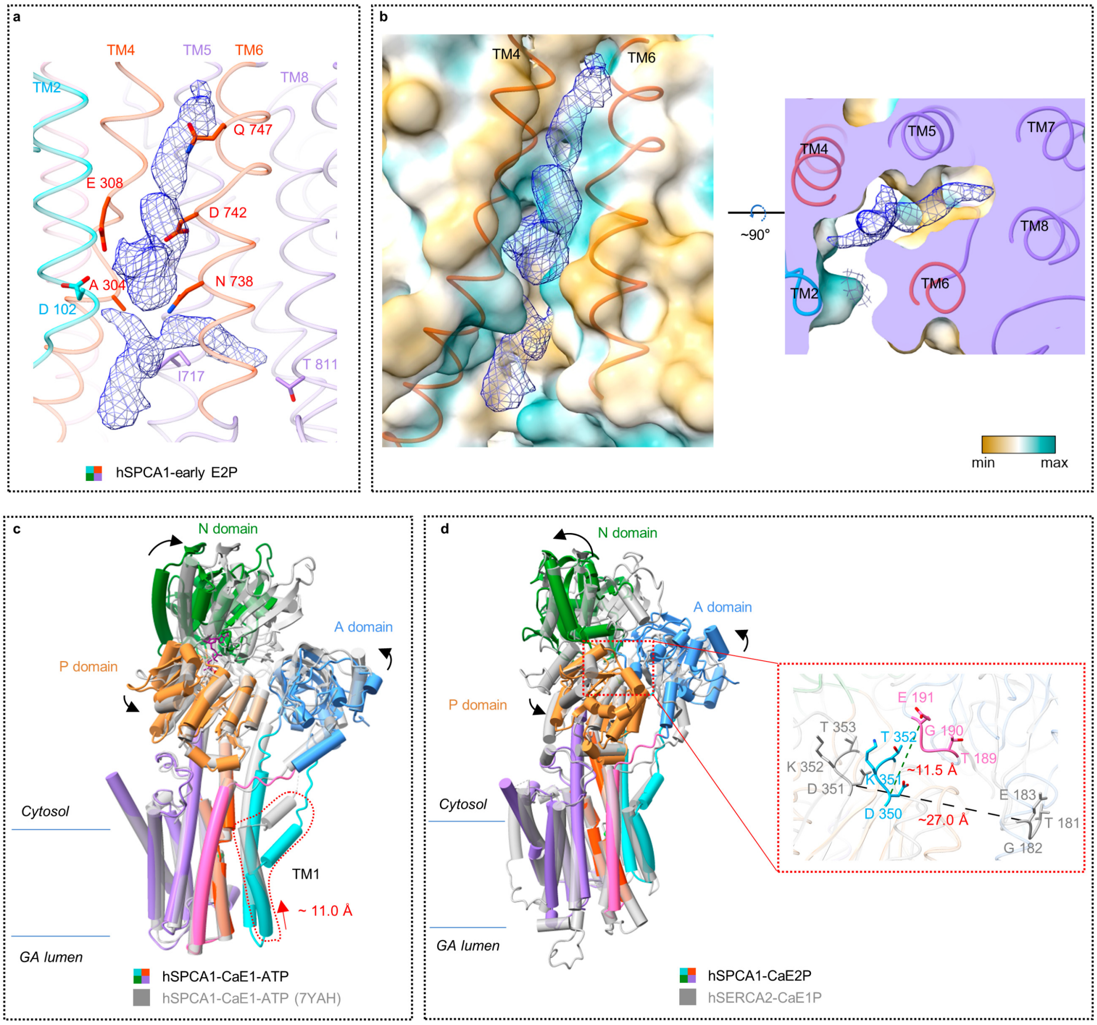

Supplementary information, Fig. S7. Structural analysis of hSPCA1. a, The large cryo-EM density (deep blue mesh, contoured at 11.0 sigma) which appears in the lumen-facing cavity of hSPCA1 in the early E2P state. **b,** Hydrophilic and hydrophobic analysis of the density environment of hSPCA1 in the early E2P state. The surface is shown with coloring ranging from dark cyan (most hydrophilic) to white (intermediate) to dark goldenrod (most lipophilic). **c**, Structural comparison of hSPCA1 in our CaE1-ATP state (colored) and the reported CaE1-ATP state (dark gray, PDB: 7YAH). The red text (~11.0 Å) shows the distance from the Cα of Leu96 in our CaE1-ATP state to the Cα of Leu96 in the reported CaE1-ATP state. **d,** Structural comparison of hSPCA1 in the

CaE2P state (colored) and hSERCA2 in the CaE1P state (dark gray, PDB: 7W7T). The black dashed line and green dashed line in the right panel show the distance from the Cα of Asp351 to the Cα of Glu183 of hSERCA2 in the CaE1P state, and the distance from the Cα of Asp350 to the Cα of Glu191 of hSPCA1 in the CaE2P state.
