# Supplementary material for: Structure and transport mechanism of the human calcium pump SPCA1
Source: Cell Res. 2023 May 31;33(7):533–45. doi: 10.1038/s41422-023-00827-x (PMC10313705; doi:10.1038/s41422-023-00827-x)
Supplement: Supplementary file 8 — Supplementary information, Table S1 [file 41422_2023_827_MOESM8_ESM.pdf]

	CaE1	CaE1-ATP	CaE1P-ADP	CaE2P	Early E2P	E2~P
PDB	8IWP	8IWR	8IWW	8IWS	8IWT	8IWU
EMDB	EMD-35776	EMD-35777	EMD-35781	EMD-35778	EMD-35779	EMD-35780
**Data collection and processing**						
Magnification	105,000 x					
Voltage (kV)	300					
Microscope	Titan Krios G3i					
Detector	K3 Summit					
Electron exposure (e^{-}/Å^2)	50					
Defocus range (μm)	1.0-1.6					
Pixel size (Å)	0.84					
Symmetry imposed	C1					
Micrographs	8208	8533	4719	8287		2454
Initial particle images (no.)	1,140,264	844,674	597,902	1,226,814		194,240
Final particle images (no.)	225,387	195,840	88,366	151,507	129,161	58,486
Map resolution (Å)	3.59	3.52	3.71	3.42	3.25	3.31
FSC threshold	0.143	0.143	0.143	0.143	0.143	0.143
B-factor applied (Å^2)	-132.1	-107.4	-110.1	-126.2	-106.9	-90
**Model building and refinement**						
Model composition						
Non-hydrogen atoms	6861	6836	6815	6809	6816	6836
Protein residues	896	893	890	890	891	893
Ligands	CA: 1	CA: 1 ACP: 1 MG: 1	CA: 1 ADP: 1 ALF: 1	CA: 1 BEF:1 MG: 1	BEF: 1 MG: 1	ALF: 1 MG: 1
R.m.s deviations						
Bond lengths (Å)	0.003	0.003	0.003	0.005	0.003	0.003
Bond angles (°)	0.687	0.596	0.762	0.672	0.542	0.575
Validation						
MolProbity score	1.83	1.77	1.97	1.72	1.56	1.69
Clashscore	8.9	8.78	13.36	7.74	7.87	7.7
Rotamer outliers (%)	0.39	0.26	0.13	0.52	0.26	0.13
Ramachandran plot						
Favored (%)	94.97	95.74	95.17	95.72	97.3	96.07
Allowed (%)	4.92	4.26	4.83	4.28	2.47	3.82
Outliers (%)	0.11	0	0	0	0.22	0.11

Supplementary information, Table S1. Data collection, processing, model refinement, and validation.
