# Supplementary material for: Structure and transport mechanism of the human calcium pump SPCA1
Source: Cell Res. 2023 May 31;33(7):533–45. doi: 10.1038/s41422-023-00827-x (PMC10313705; doi:10.1038/s41422-023-00827-x)
Supplement: Supplementary file 12 — Supplementary information, Video legend [file 41422_2023_827_MOESM12_ESM.pdf]

Supplementary information, Video S1. Trajectory of the MD simulation of hSPCA1 in the CaE2P state, showing that Ca^{2+} (green sphere) is sealed. The green mesh represents the average density of the water molecules observed in the MD simulation.

Supplementary information, Video S2. Trajectory of the MD simulation of hSPCA1 in the early E2P state, showing the potential Ca^{2+} (green sphere) release pathway. The green mesh represents the average density of the water molecules observed in the MD simulation.

Supplementary information, Video S3. Conformational cycle of hSPCA1 during Ca^{2+} transport. The cytosolic Ca^{2+} arrives at the Ca^{2+}-binding site through the hydrated cytosol-facing cavity (CaE1). The binding of ATP will induce the rotation of domain N with ~57.0° (CaE1-ATP). Next, the hydrolysis of ATP will phosphorylate Asp350 and yield the ADP molecule (CaE1P-ADP). A compact headpiece and Ca^{2+} occluded in the Ca^{2+} binding site will be generated after the ADP release (CaE2P). Subsequently, TM4L and TM6 move apart to disrupt the Ca^{2+}-binding site, generating a sufficiently large lumen-facing cavity to release Ca^{2+} (early E2P). Then, the movement of TM6 toward TM4 will close the lumen-facing cavity (E2P, PDB: 7YAM). In the following dephosphorylation process, the A and N domains will move toward the P domain (E2~P). Finally, hSPCA1 will rearrange to have a loose headpiece, with TM1-TM2 moving back toward the luminal leaflet of the lipid bilayer.
